# Template-Assisted Crystallization Behavior in Stirred Solutions of the Monoclonal Antibody Anti-CD20: Probability Distributions of Induction Times

**DOI:** 10.1021/acs.cgd.1c01324

**Published:** 2022-05-05

**Authors:** Charline J. J. Gerard, Maria L. Briuglia, Nazer Rajoub, Teresa F. Mastropietro, Wenqian Chen, Jerry Y. Y. Heng, Gianluca Di Profio, Joop H. ter Horst

**Affiliations:** † EPSRC Centre for Innovative Manufacturing in Continuous Manufacturing and Crystallisation, Strathclyde Institute of Pharmacy and Biomedical Sciences, Technology and Innovation Centre, University of Strathclyde, 99 George Street, Glasgow, G1 1RD, U.K.; ‡ Consiglio Nazionale delle Ricerche (CNR), Istituto per la Tecnologia delle Membrane (ITM), Via P. Bucci, cubo 17/C, I-87036, Rende, Cosenza, Italy; § Department of Chemical Engineering, Imperial College London, South Kensington Campus, London, SW7 2AZ, U.K.; ∥ SMS Laboratory EA 3233, Place Emile Blondel, University of Rouen-Normandie, CEDEX, F-76821 Mont Saint Aignan, France

## Abstract

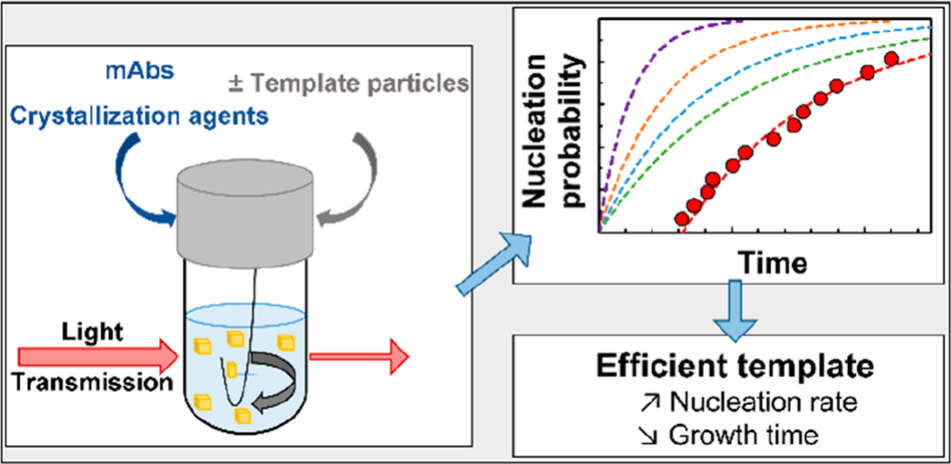

We present a method to determine the template crystallization behavior of proteins. This method is a statistical approach that accounts for the stochastic nature of nucleation. It makes use of batch-wise experiments under stirring conditions in volumes smaller than 0.3 mL to save material while mimicking larger-scale processes. To validate our method, it was applied to the crystallization of a monoclonal antibody of pharmaceutical interest, Anti-CD20. First, we determined the Anti-CD20 phase diagram in a PEG-400/Na_2_SO_4_/water system using the batch method, as, to date, no such data on Anti-CD20 solubility have been reported. Then, the probability distribution of induction times was determined experimentally, in the presence of various mesoporous silica template particles, and crystallization of Anti-CD20 in the absence of templates was compared to template-assisted crystallization. The probability distribution of induction times is shown to be a suitable method to determine the effect of template particles on protein crystallization. The induction time distribution allows for the determination of two key parameters of nucleation, the nucleation rate and the growth time. This study shows that the use of silica particles leads to faster crystallization and a higher nucleation rate. The template particle characteristics are shown to be critical parameters to efficiently promote protein crystallization.

## Abstract 2

The probability distribution of induction times gives reliable data on the crystallization behavior. This method is applied to the crystallization of pharmaceutical mAbs in the presence of various porous silica particles. The influence of these templates on the nucleation rate and the growth time of Anti-CD20 crystals is studied to identify the most efficient template to enhance the crystallization of this biopharmaceutical.

## 1 Introduction

In recent years, the use of biotherapeutics has increased significantly. Since 2015, this trend is accelerating, with an increasing number of new approvals each year, more than half of them being monoclonal antibodies (mAbs).^1^ For example, Anti-CD20 mAb, also known as Rituximab and sold under the brand name Rituxan or Mabthera, is used to treat certain autoimmune diseases and types of cancer.^2,3^ Unfortunately, the production and manufacturing of therapeutic proteins are still expensive. According to the National Institute for Health and Care Excellence (NICE, UK), in 2009 the total cost of Rituximab was £10128 per course, corresponding to six cycles of treatment. Particularly, the separation and purification of proteins are often achieved chromatographically, an expensive process.^4^ For example, most of the purification costs of mAbs (50 to 80%) are due to affinity chromatography, mainly because of the protein A resin cost.^5,6^ An analysis of 10 pharmaceutical companies by Boston Consulting Group revealed the average production cost per pack was around $5 for small molecules and $60 for biologics. This results in a daily dose of a biological drug on average being 22 times more expensive than that of a small molecule.^7^

Crystallization is a relatively easy and cost-effective process for organic pharmaceuticals manufacturing,^8^ as it allows purification, separation, and solidification of the compounds in one step, reaching purities close to 100%.^9^ Moreover, protein crystals show improved stability and conservation over time compared to amorphous formulations.^10^ In addition, protein crystals are promising injectable controlled-release systems.^11^ Crystallization is widely applied to small organic pharmaceuticals^12^ and to several commercially available proteins, such as elastase,^13^ proteases,^14^ ovalbumin^15^ and lipase.^16^ However, it is still not extensively implemented for biopharmaceuticals, and insulin^17^ remains one of the few being produced through crystallization.^18^ Indeed, even though the mechanisms involved are similar to small organic pharmaceuticals, protein crystallization development remains intrinsically complex and trial-and-error based. This is due to the large size, structural complexity and flexibility of the proteins, and the difficulty in optimization of the complex multicomponent mixtures in which the crystallization occurs.^19^

Control and enhancement of protein crystallization are crucial to achieve cost-efficient large-scale protein production. The first step of crystallization is nucleation, i.e., the formation of new crystalline nuclei, which grow out to larger sizes in the remainder of the process. Homogeneous nucleation (HON) takes place in the bulk of a clear solution at high supersaturation, while heterogeneous nucleation (HEN) is induced by the presence of foreign particles in the solution onto which the nuclei can preferentially form at lower supersaturations.^20^ Effective heterogeneous particles lower the energy barrier for nucleation and therefore increase the nucleation rate or allow nucleation at lower supersaturation so that higher-quality nuclei are formed, and their growth occurs under milder conditions.^21^ Thus, a promising way to enhance and control protein crystallization is by template-induced heterogeneous nucleation (template crystallization).^22^ Specifically, porous silica materials have been shown to be effective: they are reported to increase the crystal size and quality,^23^ increase the nucleation rate,^24^ and decrease the time required for protein crystals to nucleate.^25^ The pores of the template particles play a key role affecting nucleation. The most efficient pore size for a given protein is reported to be ∼2–10 times its radius of gyration *R*_g_(26,27) in order to stabilize the nucleus,^28,29^ which has been shown to contain roughly 1–10 biomolecules, depending on the supersaturation.^30^ Therefore, well-designed template particles promote the nucleation of proteins, as has been done to separate specific proteins through template crystallization from a binary protein solution.^31^

Usually crystallization-based purification, separation, and solidification processes involve batch crystallization under stirred conditions.^8^ However, a suitable method to study template crystallization behavior of proteins under these conditions is still needed, as protein crystallization is often achieved in stationary vapor diffusion drops. We thus aim to develop a method to determine the template crystallization behavior of proteins that combines small volume batch experiments for raw material saving, stirring conditions to mimic larger-scale processes, a statistical approach to account for the stochastic nature of nucleation, and accurate control over the crystallization conditions. We will exemplify the developed method using the mAb Anti-CD20 as the model system, as this has been shown to crystallize.^32^ Prior to the template-assisted nucleation studies, the phase diagram of Anti-CD20 mAbs is determined. Then, with a small-scale batch crystallization method, the template-assisted nucleation rate is determined using small amounts of various porous silica particles. We expect the developed method for template crystallization behavior to ease the design of template crystallization processes for mAbs and other proteins.

## 2 Materials and Methods

Anti-CD20 mAbs (*M*_W_ = 144.488 kDa, radius of gyration *R*_g_ = 5.2 nm^33^), produced in a Chinese hamster ovary (CHO) mammalian cell line, was provided by the Centre for Process Innovation (CPI) and FUJIFILM Diosynth Biotechnologies (UK) at a concentration of 9 mg/mL in stock buffer (25 mM sodium citrate, pH = 6.5, 154 mM NaCl (Sigma-Aldrich, BioXtra ≥99.5%)). The mAbs were stored in 1 mL tubes at −80 °C until used.

### 2.1 Anti-CD20 Solution

The homogeneity of Anti-CD20 mAb solutions was measured by dynamic light scattering (DLS) with a Zetasizer Nano ZS instrument (Malvern) equipped with a 4 mW He–Ne laser at 632.8 nm. Anti-CD20 mAb samples at ∼0.1 mg/mL in crystallization buffer (100 mM HEPES, Sigma-Aldrich ≥99.5%, pH 7.4) were filtered on a 0.22 μm filter (Anotop 10, Whatman) before measurement and placed in ultralow volume quartz cuvettes (ZEN 2112, optical path 3 mm, Malvern), to measure the protein hydrodynamic diameter. The light intensity and its time autocorrelation function were measured at a scattering angle of 173°. All measurements were performed at 20 °C after 2 min of equilibration using automatic time settings. The Anti-CD20 mAbs solutions have been shown to be homogeneous in the crystallization buffer, without aggregates or fragments. The average hydrodynamic diameter is 10.9 nm, i.e., average hydrodynamic radius *R*_h_ = 5.5 nm. From these measurements, we assume the size of the Anti-CD20 molecules is 9–13 nm.

**Figure 1:**
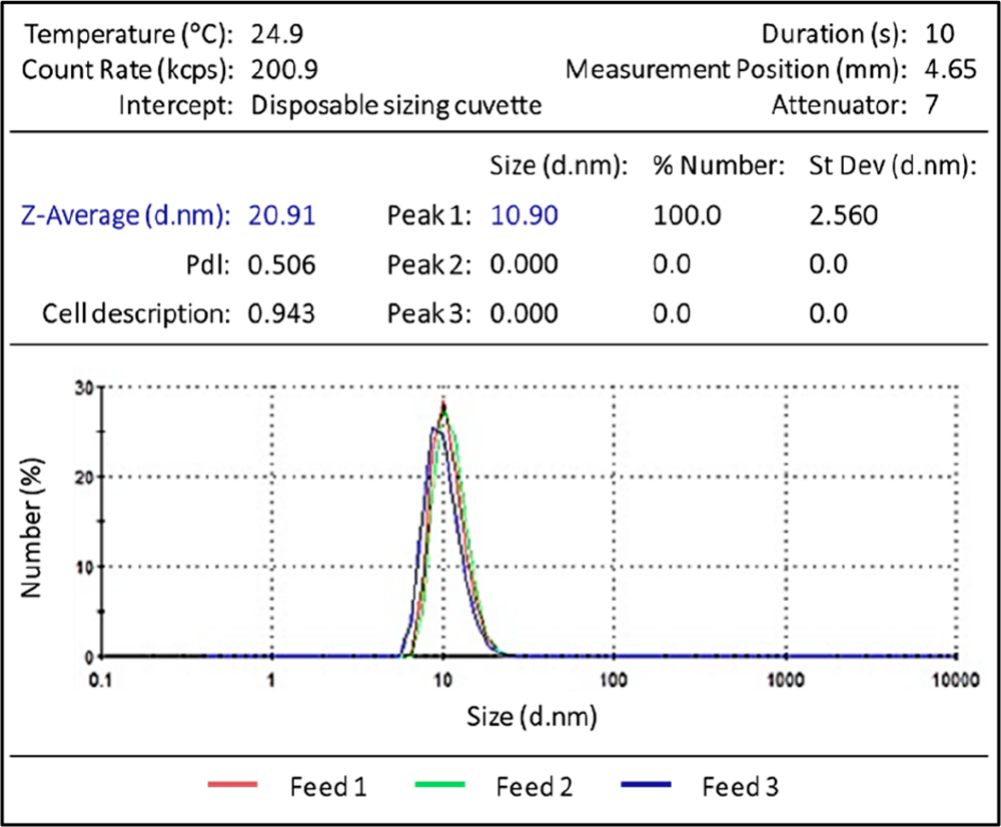
DSL measurement of Anti-CD20 mAbs hydrodynamic diameter in the crystallization buffer.

For crystallization trials, 10 tubes of stock mAbs were thawed for roughly 1 h at room temperature. The stock buffer of the mAbs solution was exchanged by the crystallization buffer (100 mM HEPES, Sigma-Aldrich ≥99.5%) using Amicon Ultra-15 centrifugal filter units (Ultra-4, cutoff 3 kDa, Merck-Millipore). The resulting solution was adjusted to a pH of 7.4 using NaOH solution, Sigma-Aldrich, BioXtra ≥98%). Three centrifugation cycles (Eppendorf 5810 R) of 15 min at 7000 rpm and 6 °C were run, adding each time 5 mL of the crystallization buffer to 1 mL of mAbs solution. The Anti-CD20 concentration was determined by absorbance measurements at 280 nm using a UV–vis spectrophotometer (Chirascan, Applied Photophysics) and a molar extinction coefficient ε^280^ = 237 380 L·mol^–1^·cm^–1^ for Anti-CD20. Typically, the final mAbs concentration was nearly 100 mg/mL, and less than 2% of the stock buffer remained in final mAbs solution. The protein solution was used immediately after preparation.

### 2.2 Salt Solution with Template Particles

The salt solutions were prepared dissolving 10%, 11% or 12% (v/v) of PEG-400 (Sigma-Aldrich BioUltra) and 1, 1.1, or 1.2 M (respectively) of anhydrous Na_2_SO_4_ (Sigma-Aldrich, puriss. p.a. ≥ 98%) in the crystallization buffer. All the solutions were prepared using distilled water (Milli-Q gradient, Millipore SAS).

The controlled porous glass (CPG) particles were purchased from Sigma-Aldrich (120–200 mesh). Other templates, core–shell nanoparticles (CS), amorphous silica (AS), and mesostructured silica (MS) were prepared following guidelines from the literature.^34^ The template particle features are listed in Table 1, and TEM pictures of the templates are shown in Figure S1. The pore shape of all particles is assumed to be spherical.

**Table 1:** Mesoporous Silica Template Features

name	particle shape	particle size distribution	pore size (nm)
CS	spherical	250 nm	4
CPG	irregular	74–125 μm	50
AS	tubular	600 × 150 nm	4
MS	hollow sphere	40 nm	40

The templates were suspended in the salt solution containing the crystallization agents (Na_2_SO_4_–PEG-400), and the resulting suspension was sonicated for 1 h at room temperature to ensure template particle dispersion in the solution and to prevent their aggregation. The template concentration in that solution was calculated in such a way that the final template concentration in the crystallizing solutions was 0.5 mg/mL.

### 2.3 Microbatch Crystallization Experiments

Microbatch crystallization experiments to determine the phase diagram and measure the induction times were run using the CrystalBreeder (Technobis). This setup contains 32 stirred reactors of 200 μL each monitored through light transmission, with accurate control over the temperature. Crystallization samples were prepared by mixing the salt solution, either with or without template particles, and the mAbs solution, directly in vials. The vials are then placed in the CrystalBreeder at 20 °C, and the transmission of light through each sample is recorded in time. At the beginning of the experiment, before protein crystallization occurred, the solution is clear, and light transmission is 100%, even with solutions containing template particles as the detection limit is slightly 0.5 mg/mL of particles in the suspension, as tested in separate experiments. When crystallization occurs in the reactor, the light transmission decreases due to a large suspension density. The time period between the start of the experiment and the time at which the light transmission started to decrease from 100% was taken as the induction time for that sample composition. All the crystallization experiments were run at 20 °C using a stirring rate of 700 rpm.

#### 2.3.1 Phase Diagram

Crystallization solutions were prepared mixing a specific amount of the salt solution with an amount of the mAbs solution in the vials. It was previously described that Na_2_SO_4_–PEG-400 solutions can undergo a liquid–liquid phase split (LLPS) at concentrations above 1 M of Na_2_SO_4_ and 11% (w/w) of PEG-400 in pure water solutions.^35^ In the case of a LLPS, the crystallization medium is not homogeneous, which affects the conditions of crystallization and can prevent nucleation for several hours.^36^ Moreover, the impurities often concentrate in the solute-rich phase, leading to high impurity integration in the crystals.^37^ Therefore, conditions are chosen to avoid an instant spontaneous phase split. A large range of Na_2_SO_4_ and PEG-400 concentrations (respectively 0.5–1.1 M and 5–11% *v/v*) in the mixed samples is used to ensure the identification of suitable crystallization conditions. The pH is set at 7.4, which is within the range in which Anti-CD20 crystals have been previously obtained.^32^ In order to reduce the amount of Anti-CD20 required, typically, only concentrations below 35 mg/mL are used.

All the crystallization experiments were monitored for 48 h, after which it was recorded whether a suspension or a clear solution was present in the vials. Conditions that did not lead to crystal formation during this period were stated as conditions in which no nucleation occurs, even though a longer time might have led to crystallization in some vials. A sample from each vial was then observed using a microscope (Leica DM6000M) to confirm the occurrence of protein crystallization and to discriminate conditions leading to crystallization from those leading to precipitation. A precipitate is generally believed to be a poor and impure product formed due to a too high supersaturation.

#### 2.3.2 Induction Time Measurements

Induction time measurements were run for at least 24 h. Crystallization conditions were chosen as 25 mg/mL of Anti-CD20, 0.75 M of Na_2_SO_4_, 7.5% of PEG-400 (resulting in a supersaturation of around *S* = 1.25) in the presence or absence of 0.5 mg/mL of template, at 20 °C under stirring conditions (700 rpm). Note that a concentration of 0.5 mg/mL of template particles does not influence the transmission of light through the vials. The crystallization conditions were chosen from the phase diagram, to ensure nucleation within a reasonable time period and to avoid precipitation. Each condition was reproduced 16 times without template and with CPG and CS particles, and 32 times with MS and AS particles. Induction times were determined as the time period from the creation of supersaturation to the point in time at which the transmission of light through the vial started to decrease. Each point on the graphs corresponds to an independent experiment.

## 3 Results

### 3.1 Phase Diagram of Anti-CD20

Anti-CD20 mAbs has previously been crystallized by the vapor diffusion method at 20 °C, using 20–60 mg/mL of Anti-CD20, 0.6–1.5 M of Na_2_SO_4_, and 8–12% w/v (weight/volume) of PEG-400, with 0.1 M of HEPES buffer at pH 6.8–8.1.^32^ Typically, small needle-like crystals between 10 and 50 μm long appeared after 2–3 days. Anti-CD20 crystals were obtained within 12 h when using the batch method with 60 mg/mL of mAbs, 770 mM Na_2_SO_4_, and 24% w/v PEG-400 in 400 μL to 1 mL vials. To date, no phase diagram or solubility of Anti-CD20 has been reported.

**Figure 2:**
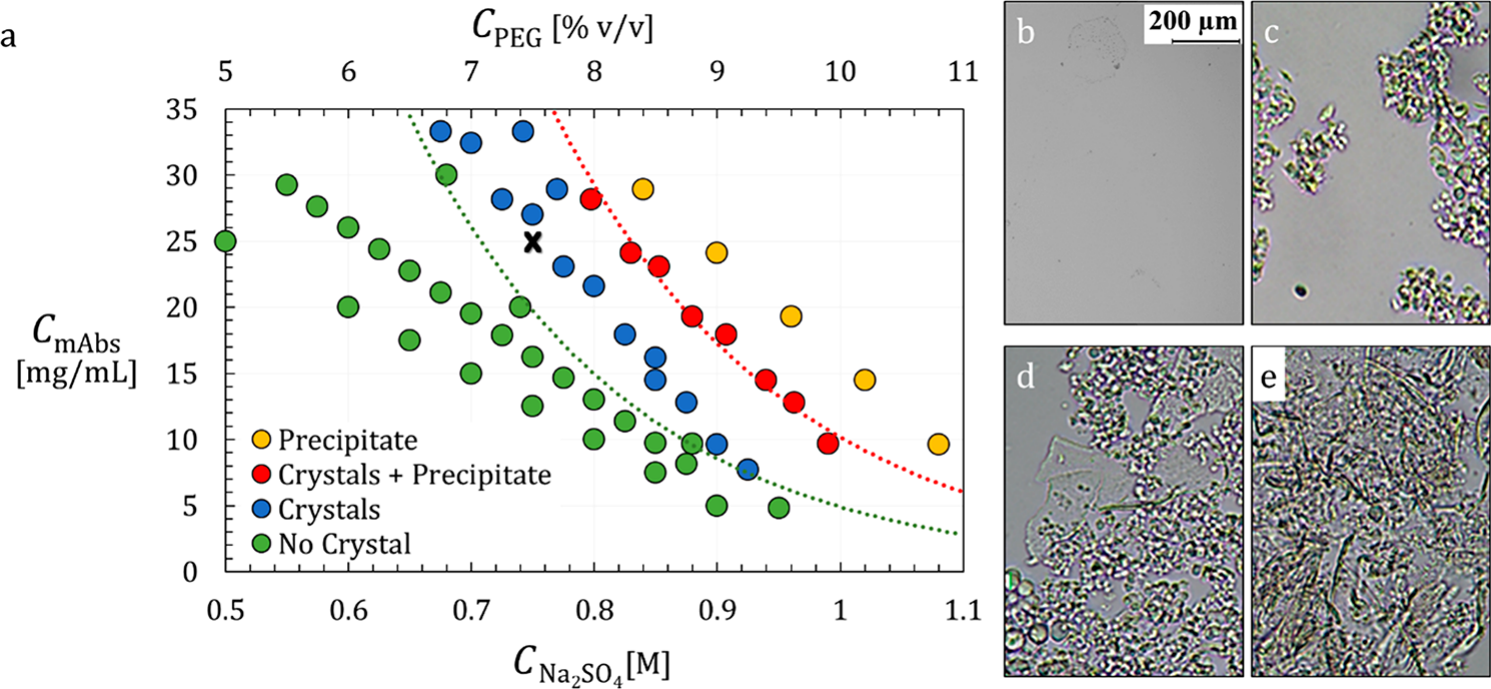
(a) Phase diagram of Anti-CD20 mAbs at 20 °C, using 1% PEG (v/v) for 0.1 M Na_2_SO_4_ solutions as a crystallization agent, at pH = 7.4. The points are colored by their crystallization result after 48 h under stirring conditions without templates. The dash lines are a guide for the eyes, the green line is the metastable zone limit, and the red line estimates the precipitation line. The black cross highlights the crystallization condition chosen to study the crystallization behavior of Anti-CD20. On the right, pictures of (b) a clear solution, (c) Anti-CD20 crystals, (d) Anti-CD20 crystals and precipitate, and (e) precipitate. Precipitate and crystals are distinguished visually.

To estimate the phase diagram, the crystallization behavior of Anti-CD20 as a function of Na_2_SO_4_ and PEG-400 concentrations is determined. Figure 2a shows the crystallization ability of Anti-CD20 as a function of Na_2_SO_4_/PEG-400 and protein concentration. Indeed, many conditions lead to mAbs crystallization within 48 h. The obtained crystals are quite small; the largest are roughly 20 μm long (Figure 2c), which is consistent with previously obtained Anti-CD20 crystals.^32^ Their size decreases when using higher protein and PEG-400/Na_2_SO_4_ concentrations, i.e., when approaching the precipitation zone. The crystallization zone (area with the blue points in Figure 2a, crystals shown in Figure 2c) is delimited on one side by the metastable zone limit (green dashed line in Figure 2a), below which nucleation does not occur spontaneously within 48 h (as shown in Figure 2b). On the other side, it is delimited by the precipitation line (red dashed line on Figure 2a), beyond which a precipitate is formed. In that area, the crystal habit is not well-defined, and the resulting crystals are very small (Figure 2d,e).

The experiments to establish the crystallization ability were run only for 48 h, in order to keep a reasonable crystallization time. Indeed, the induction time of protein crystallization can be very long.^19,38^ Therefore, some experiments were presumably stopped before crystallization could occur. Thus, the solubility line of Anti-CD20 has not been determined, but rather the metastable zone limit below which no crystallization occurs within 48 h has been determined. Therefore, the actual solubility line would be positioned below the metastable zone limit. This means that Figure 2a represents a kinetic phase diagram.

This kinetic phase diagram does allow the identification of suitable crystallization conditions in a stirred batch for further template crystallization behavior studies for Anti-CD20. We chose conditions that ensure crystallization in the absence of template particles within 24 h, to keep a reasonable experiment time, while preventing the appearance of a precipitate, to avoid too fast and uncontrolled nucleation so that the effect of templates on the crystallization behavior would be apparent. Therefore, we chose the condition to be at a concentration of 25 mg/mL of Anti-CD20, 0.75 M of Na_2_SO_4_, and 7.5% of PEG-400 (Figure 2a, black cross).

### 3.2 Nucleation Behavior of Anti-CD20 in the Absence of Template Particles

Nucleation is of a stochastic nature; the number of crystals that appear in a certain volume at a certain time is a random variable. In other words, identical experimental conditions will lead to different nucleation rates and induction times.^39^ The induction time is the time required for the crystals to be detected in an initially clear solution at constant supersaturation. This stochastic nature of nucleation has been exploited for studying the crystallization behavior of organic molecules in batch under stirring conditions, by measuring the induction times of several crystallization trials under identical conditions (composition, temperature, stirring rate, and volume).^40,41^

**Figure 3:**
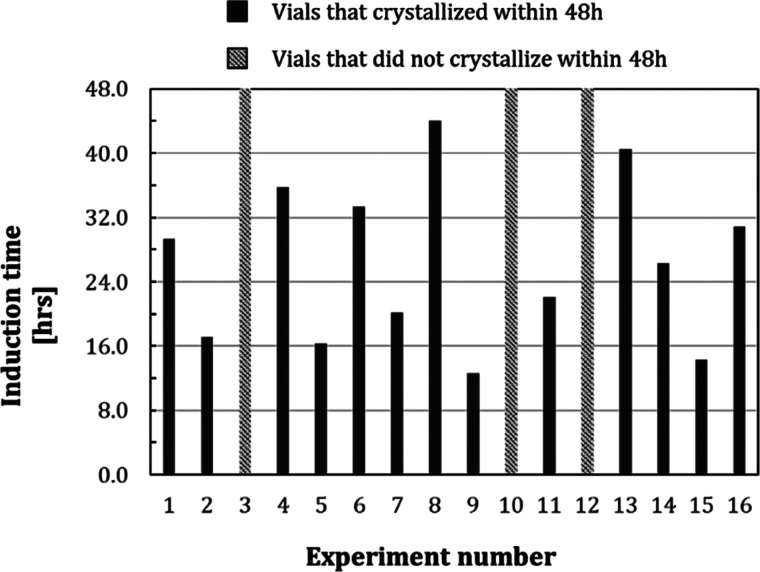
Induction times of 16 identical and independent Anti-CD20 crystallization trials for solutions without template. The vials that did not crystallize within 48 h are shown with patterned filling. Experimental conditions are 25 mg/mL of Anti-CD20, 0.75 M of Na_2_SO_4_, and 7.5% of PEG-400, pH = 7.4, 20 °C, in 200 μL stirred vials.

The induction times of Anti-CD20 mAbs crystallization is first measured without the addition of any heterogeneous particles, in 16 identical experiments. A wide range of induction times is found, reflecting the stochastic nature of crystallization. Indeed, the minimum induction time measured is 12.3 h (experiment 9 in Figure 3), while for three experiments, no crystals are detected within 48 h. The median induction time is 26.3 h, which shows that the crystallization process of Anti-CD20 is very slow under the probed conditions.

**Figure 4:**
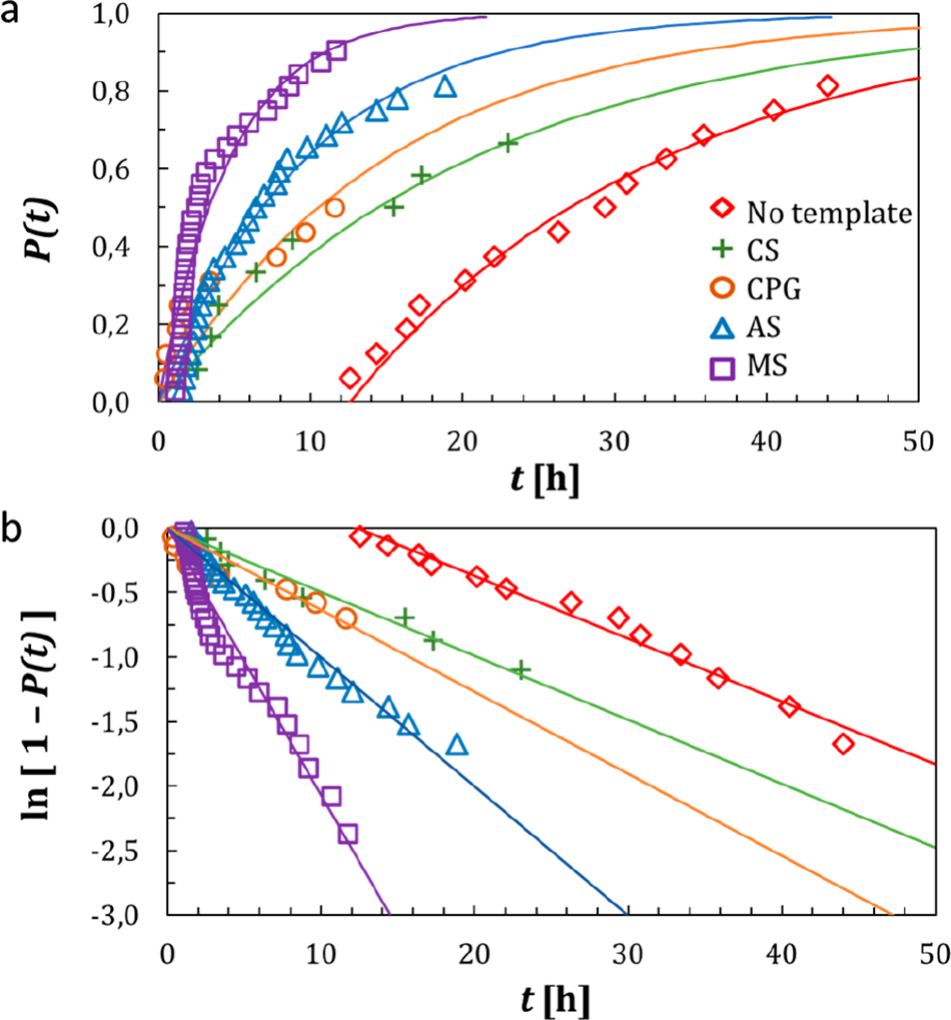
(a) Probability distribution *P*(*t*) of the induction times and (b) linear regression of the induction times using eq 3 to calculate nucleation rate *J* and growth time *t*_g_ for Anti-CD20 crystallization without template (red diamond) and with 0.5 mg/mL of each template: CS (green cross), CPG (orange circle), AS (blue triangle), and MS (purple square).

For *M* independent experiments, the experimental induction time probability *P*(*t*) to measure an induction time *t* is defined as
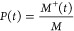
1where *M* is the total number of identical experiments (here 16), and *M*^*+*^(*t*) is the number of experiments in which crystals are detected at time *t*. For instance, the probability at *t* = 48 h, where 3 of the 16 vials still show clear solutions, is *P*(*t*) = 13/16 = 81%. For each measured induction time (Figure 3), the corresponding *P*(*t*) is calculated, and the *P*(*t*) values are then plotted against the time *t* (Figure 4a). The probability distribution of induction times so obtained is well-described by the probability distribution function described by^40^
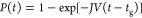
2which can be linearized as
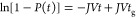
3where *J* is the nucleation rate, *V* is the volume of the vial (here, 200 μL), *t* is the induction time, and *t*_g_ is the growth time, which accounts for the delay time between nucleation of the first crystal and detection of the suspension.

Thus, if a substantially large number of statistically independent and identical experiments is run, this method allows the calculation of the nucleation rate *J* and growth time *t*_g_ of crystals by fitting the experimental probability distribution with the linearized distribution function in eq 3. Here, the nucleation rate *J* and growth time *t*_g_ are determined from respectively the slope and intercept of the line when plotting ln[1 – *P*(*t*)] against time *t*.

The obtained experimental induction time probability distribution of Anti-CD20 without templates is shown by the red line in Figure 4a,b, and the resulting *J* and *t*_g_ values are summarized in Table 2. As the batch method is used for crystallization experiments, the supersaturation is generated at *t* = 0 s. Still, more than 12 h are required for the first crystals to be detected in the solution. This is not only due to the slow nucleation rate of 244 crystals·L^–1^·h^–1^ but also because of the very high growth time *t*_g_ of 12.3 h (Table 2), which represents the time needed for the nucleated crystals to be detected.

**Table 2:** Nucleation Rate and Growth Time of Anti-CD20 Crystals, in the Presence of Various Mesoporous Silica Templates, Determined by Fitting eq 3 to the Data in Figure 4^a^

template	*J* [#·L^–1^·h^–1^]	*t*_g_ [h]	*R*^2^
none	244 ± 21	12.3 ± 0.8	0.980
CS	249 ± 20	≤0.7	0.957
CPG	318 ± 70	≤1.2	0.690
AS	501 ± 20	≤0.7	0.972
MS	1037 ± 55	≤0.6	0.950

a Crystallization conditions are Anti-CD20 25 mg/mL, Na_2_SO_4_ 0.75 M, PEG-400 7.5%, template particles 0.5 mg/mL, pH 7.4, 20°C, in 200 μL stirred vials.

The Anti-CD20 crystals growth rate can be roughly estimated to be 0.7 nm·s^–1^, considering crystals grow to a size of about 30 μm in 12 h. This growth rate is much slower than that for small organic molecules but also slower than that for lysozyme, the most commonly model protein used for crystallization studies, which can be estimated between 2.5 and 25 nm·s^–1^^42,43^ depending on the crystallization conditions. However, lysozyme is often used as a model protein because it crystallizes well and relatively fast, which is not completely representative of common protein crystallization behavior. Indeed, nucleation and crystal growth of complex and flexible biomacromolecules, such as mAbs, are much slower than that of smaller molecules.^38^ Their considerably larger size, lower diffusivity, and weaker association tendencies compared with small molecules or ions as well as the lower probability of incorporation of an incoming macromolecule into a growth step make their crystal nucleation and growth kinetics generally 2–3 orders of magnitude slower than that of small molecules.^19^

### 3.3 Template-Assisted Heterogeneous Nucleation Behavior of Anti-CD20

The same method is then applied for a template-assisted heterogeneous nucleation study, by measuring series of induction times of Anti-CD20 solutions in the presence of mesoporous silica templates, all other parameters being identical. It must be noted that this method is not applicable to turbid solutions, as such conditions would disturb the transmission of light through the solution sample. An amount of up to 0.5 mg/mL of template particles did not show any effect on the transmission of light through the sample. The resulting probability distribution of induction times of nucleation with and without templates is shown in Figure 4, and nucleation rate *J* and growth time *t*_g_ are given in Table 2.

First, Figure 4 shows a clear reduction in induction time for Anti-CD20 crystallization when templates are added to the crystallization solution. Indeed, with every template, Anti-CD20 crystallization started within the first hour, while without it, more than 12 h were required. For example, with MS template particles (Figure 4 purple line), the first crystallization occurs after 1 h 45, and after 12 h only three vials did not lead to crystallized mAbs. The median crystallization time is 2 h, while without a template the median crystallization time is 26 h. This decrease is reflected by the much lower *t*_g_ values for crystallization with templates, shown in Table 2, where *t*_g_ is reduced from 12.3 h without template to less than 42 min with template particles.

Even though all the porous silica particles efficiently decrease the growth time *t*_g_, and thus the induction time, they are not all efficient in increasing the nucleation rate. The CS particles, notably, result in the same nucleation rate of Anti-CD20 crystals as in the absence of templates (both a little less than 250 crystals·L^–1^·h^–1^). Also, the slight increase in the nucleation rate in the presence of CPG particles is perhaps not significant. Conversely, AS particles double the nucleation rate to 500 crystals·L^–1^·h^–1^. The most efficient template particles to increase the nucleation rate of Anti-CD20 crystals are MS particles, which give a nucleation rate more than four times higher than the nucleation rate without added particles (1037 crystals·L^–1^·h^–1^).

These results show that heterogeneous templates can be efficient catalysts to accelerate the crystallization process of complex biomacromolecules such as Anti-CD20. However, as expected, the effect of templates on the nucleation rate is particle-dependent, and therefore the template particle used must be optimized to obtain the preferred nucleation behavior. The induction time distribution measurement method described here is an efficient method for this.

## 4 Discussion

The determination of the experimental probability distribution of induction times is an accurate method to study the effect of a specific parameter, such as the effect of template particles, on the crystallization behavior in systems, even complex crystallizing systems involving monoclonal antibodies like Anti-CD20. Two key parameters of nucleation, the nucleation rate *J* and the growth time *t*_g_, can be quickly and easily determined in conditions comparable to industrial conditions, i.e., in a stirred batch, while the amount of protein material needed remains reasonable due to the small volumes involved in the experiments. From the probability distribution of induction times under various conditions, the crystallization behavior of protein can be compared to extract the best conditions to crystallize biomolecules. In the case of Anti-CD20 mAbs, this study shows that the addition of MS template particles to the crystallization cocktail leads to the highest nucleation rate and the lowest growth time. Such particles therefore could be helpful in controlling the crystal nucleation and growth of such proteins on an industrial scale.

The probability distribution of induction times gives accurate data on crystallization that shows the stochastic behavior of nucleation in trials with identical conditions. Moreover, the use of 200 μL batch vials remains reasonable in terms of raw material required for the study, even in the case of scarce protein samples. We show here that crystals of complex biomacromolecules, such a mAbs, can be crystallized in stirred batch processes, which broadens the possible processes of industrial production of biopharmaceuticals. Being closer to reproducible industrial parameters, the crystallization process exploited here would be easier to scale-up than the vapor diffusion method. However, the crystallization process is not visually monitored by time; only the transmissivity of light through the sample is recorded, and the final crystals are observed. Therefore, important intermediate phenomena such as a liquid–liquid phase separation could be missed.

For Anti-CD20, it is interesting to note that templates affect not only the nucleation rate but also substantially the growth time. Indeed, with all templates used, Anti-CD20 crystals were detected in a large part of the samples within the first two hours, while without it, more than 12 h were required. Particularly, the growth time *t*_g_ of Anti-CD20 crystals is substantially decreased when HEN is triggered. The *t*_g_ has been shown to rely on the crystal growth rate and secondary nucleation.^44^ Template particles are not expected to directly affect these parameters. Another phenomenon might thus be involved to explain this substantial drop in *t*_g_. At high concentrations, PEG-400/Na_2_SO_4_ solutions, in the absence of protein, can lead to a liquid–liquid phase separation (LLPS).^35^ Such a LLPS has been reported to be concomitant with Anti-CD20 crystallization by the vapor diffusion method^45^ in PEG-400/Na_2_SO_4_ solutions, showing that the LLPS occurrence is closely linked to the crystallization conditions of Anti-CD20 mAbs. Authors in ref (45) suggest that the LLPS induces a local change in the Anti-CD20 mAbs conformation at the interface between the two liquid phases, which promotes protein aggregation and thus triggers the crystallization process.

Even though the crystallization conditions chosen to perform the probability distribution of induction times do not lead to an instantaneous phase split, porous template particles are known to promote the nucleation kinetics of droplets in metastable solutions regarding LLPS, just like that of crystals.^46−48^ Therefore, by triggering the LLPS, template particles could favor this local conformational change that may have an impact on crystallization kinetics. The observed drop in *t*_g_ could thus be correlated to the occurrence of the LLPS in the presence of template particles. Moreover, as crystals appear at the interface of protein-rich droplets, the higher protein concentration available in these droplets could lead to a faster growth rate, further reducing *t*_g_. It must be highlighted here that the occurrence of an LLPS could affect the light transmission through the sample. However, LLPS is induced at the surface of the particles only, or very close to them, which then would not dramatically influence the transmission of light. Moreover, previous studies on Anti-CD20 crystallization showed that when an LLPS occurs in the crystallization solution, the nucleation of mAbs crystals occurs at the same moment.^32,45^ These two phenomena being closely correlated, we assume the occurrence of one triggers the occurrence of the other, making it hard to discriminate which phenomenon is actually detected when the light transmission through the sample reduces.

The template effect on the Anti-CD20 nucleation rate is less explicit. Although the CS template particles do substantially affect the growth time *t*_g_, the nucleation rate *J* is not significantly changed. AS particles, with the same pore size of 4 nm as the CS template particles, give a higher nucleation rate (twice that of the solutions without templates). For both CS and AS templates, the pores are smaller than the hydrodynamic radius *R*_h_ of Anti-CD20 (5.5 nm); they thus are too small to trap Anti-CD20 and promote the formation of the nucleus. The optimal pore size for template-induced nucleation has been reported to be of roughly 1–5 times the molecule size to induce efficiently a local increase of supersaturation, or to stabilize strongly the nuclei.^26,27,29^ As the average size of Anti-CD20 molecules is 9–13 nm (Figure 1), this results in an optimal pore size in the size region from 9 to 65 nm. The tubular streaks we observe at the surface of the AS template particles (roughly 10 nm, Figure S1d) could favor the trapping of the molecules instead of the pores. However, they might not be optimal to stabilize nuclei, exemplified by only a slightly increased nucleation rate. On the other hand, the pore size of the CPG particles (50 nm) corresponds to the theoretical upper size limit for pores to impact the nucleation rate. This results in a small effect on the nucleation rate, but the experimental data with CPG do not fit well with the theoretical equation, as is shown by the low *R*^2^ value on Table 2, leading to a large uncertainty on *J*. Therefore, the slight increase of *J* observed in the presence of the CPG particles is not significant, and no clear conclusion can be given from our experimental data about the nucleation rate of Anti-CD20 in the presence of CPG particles. The most efficient template to increase the nucleation rate of Anti-CD20 crystals is MS, which gives a nucleation rate more than four times higher than the nucleation rate in the absence of templates. We postulate that the pore size of MS particles (40 nm, i.e., 8*R*_g_) is close to the optimal one, large enough to preferentially trap the protein molecules and induce a local higher supersaturation and small enough to stabilize the nucleus.

The pore size is thus crucial to promote nucleation: if the pores are too small, the protein molecules are not trapped, and there is no local increase of supersaturation; if the pores are too large, they do not stabilize the nucleus. On the other hand, the particle shape and surface also affect nucleation: streaks at the particles surface, if small enough, help the trapping of the biomolecules and induce local supersaturation, and hollow sphere particles gave the highest nucleation rate, as the pore accessibility by the molecules was the most favorable. Moreover, as the total mass of particles in the solution is the same in every experiment, the smallest particles give the highest surface area. Therefore, the shape of the MS template particles is highly favorable. This shows the advantages of using especially designed templates to enhance crystallization of a protein of interest as pore size and particle shape are of importance to promote nucleation.

## 5 Conclusion

We studied the template crystallization of the monoclonal antibody of pharmaceutical interest, Anti-CD20. The mAb has been crystallized in the presence of PEG-400 and Na_2_SO_4_ using the batch method under stirred conditions, and a kinetic phase diagram has been determined showing an area in which Anti-CD20 can be crystallized within 48 h. Then, using the experimentally determined probability distribution of induction times, nucleation in the absence of templates has been compared to template-assisted nucleation using mesoporous silica templates. The probability distribution of induction times, applied to protein crystallization, is shown to be an accurate method to study the effect of a specific parameter (here the presence of template particles) on the crystallization behavior. The method presented here allows investigations on the influence of key parameters, such as the pore size of template particles, on the crystallization behavior of proteins. Two key parameters of nucleation, the nucleation rate *J* and the growth time *t*_g_, can be quickly and easily determined in conditions comparable to industrial conditions, i.e., in a stirred batch, while the amount of raw protein material used remains reasonable due to the low volume experiments. From the probability distribution of induction times, the crystallization behavior of protein can be compared to extract the best conditions to grow biomolecules crystals. In the case of the pharmaceutical Anti-CD20 mAbs, this study shows that the use of silica template particles leads to faster crystallization and a higher nucleation rate. Heterogeneous nucleation with templates is thus a promising way to selectively crystallize a biopharmaceutical from a complex solution.

## Supporting Information Available

TEM images of the template particles CPG, MS, CS, and AS (PDF)
