# Supplementary figures and images for: Template-Assisted Crystallization Behavior in Stirred Solutions of the Monoclonal Antibody Anti-CD20: Probability Distributions of Induction Times
Source: Cryst Growth Des. 2022 May 5;22(6):3637–45. doi: 10.1021/acs.cgd.1c01324 (PMC9164231; doi:10.1021/acs.cgd.1c01324)

## Supplementary material

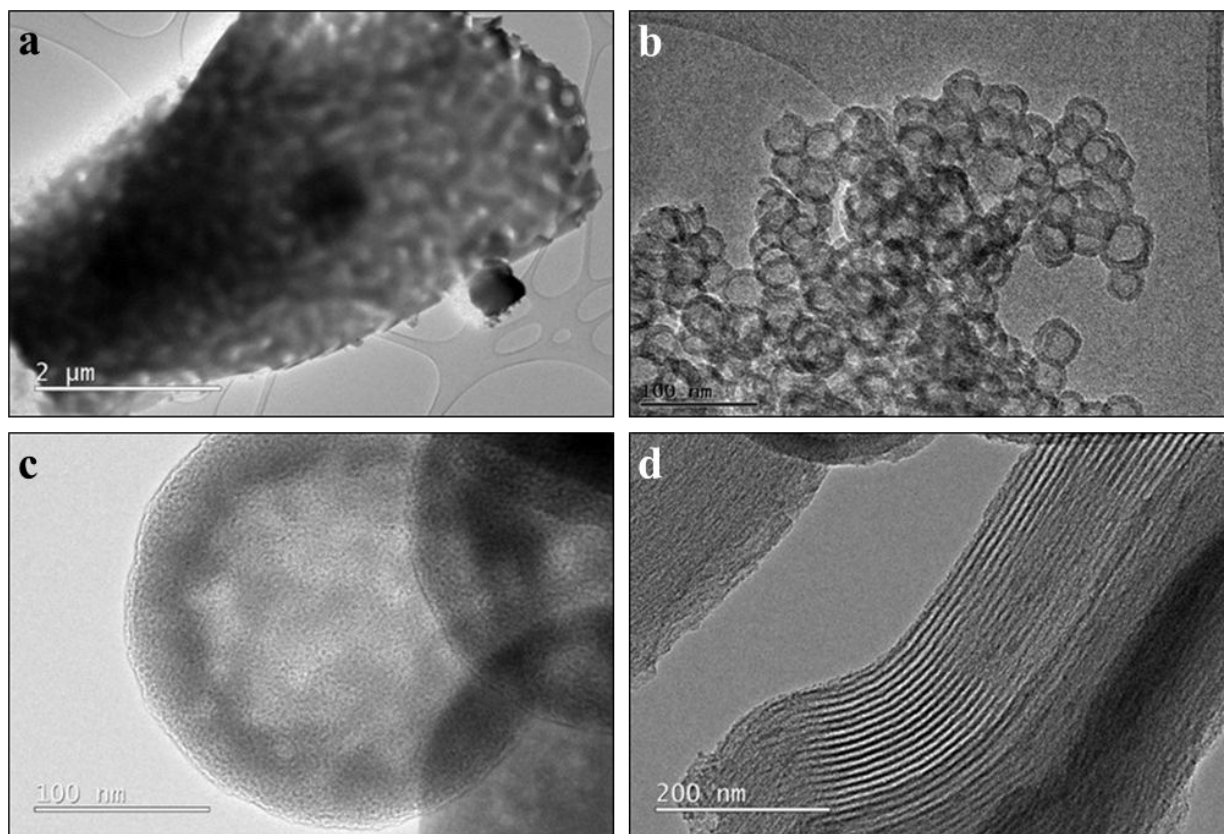

Figure S 1: TEM images of the templates (a) CPG, (b) MS, (c) CS and (d) AS.

Supplement: Supplementary file 1 — cg1c01324_si_001.pdf [file cg1c01324_si_001.pdf]
